# The Association between Type-1 Diabetes Mellitus and Risk of Depression among Saudi Patients: A Cross-Sectional Study

**DOI:** 10.3390/jpm13040654

**Published:** 2023-04-11

**Authors:** Bashair Aldossari, Abdulaziz Alhossan, Ajaz Ahmad

**Affiliations:** Department of Clinical Pharmacy, College of Pharmacy, King Saud University, P.O. Box 2457, Riyadh 11451, Saudi Arabia

**Keywords:** type-I diabetes mellitus, depression, comorbidities, Saudi population

## Abstract

Background and Aims: The importance of screening type-1 diabetic patients in Saudi Arabia is related to a high incidence rate of diabetes mellitus (DM) and the susceptibility to developing depression during or after the diagnosis. The objectives of the present study were to establish the relationship between type-1 diabetes mellitus (T1DM), depression, and depression risk among Saudi patients; estimating the prevalence and examining the relationship of depression with duration of diagnosis, the effect of glycemic control, and the presence of comorbidities. Methods: For this observational retrospective chart review, an analytical tool was used. The population of our study comprised Saudi patients with T1DM at King Khaled University Hospital, Riyadh. Data were collected from the hospital’s electronic medical records. A depression screening tool (Patient Health Questionnaire “PHQ-9”) was used to measure the depression risk of the diabetic patients, who had not been assessed before. The SPSS program was used to analyze the data. Results: The present study included 167 males (~45.75%) and 198 females (~54.25%). Patients with a normal body mass index (BMI) constituted 52%, while 21% were underweight, 19% were overweight, and 9% were obese. The investigators randomly selected 120 patients from the total of 365, and called them to assess their risk of developing depression. The results of the depression assessment were as follows: positive, 17 patients out of 22 (77.27%); negative, five patients out of 22 (22.73%). In total, 75 out of 120 (62.50%) patients were at risk of developing depression, while 45 patients out of 120 (37.50%) were not at risk of depression. There was a relationship between glycemic non-control, comorbidities with depression, and risk of developing depression in DM. The presence of complications was associated with diabetic and depressed patients, and the risk of developing depression may be increased with T1DM. Conclusions: To overcome the negative consequences of undiagnosed depression, screening for depression is recommended for patients with T1DM who have multiple comorbidities, glycemic non-control, diabetic complications, and unfavorable lifestyles, as well as those undergoing combination therapy with metformin.

## 1. Introduction

DM is one of the most widespread diseases and a leading cause of comorbidity worldwide [1]. The international Diabetes Federation states that “diabetes is one of the largest global health emergencies of the 21st century” [2]. The global prevalence is estimated to be 9.30% and this is projected to increase by 25% in 2030 and 51% in 2045 [3]. The growing and aging population are factors in the overall rise in diabetes incidence, resulting in premature mortality, disability, morbidity, and greater healthcare expenses [4]. DM is divided into various types, including T1DM, type-two diabetes mellitus (T2DM), and gestational diabetes mellitus (GDM) [5]. T1DM can be defined as an endocrine disorder characterized by insulin deficiency, usually due to autoimmune pancreatic β-cell destruction; T2DM occurs when the body is unable to use insulin properly, and GDM is a condition that pregnant women develop when their body is not able to make and use insulin properly [6]. This results in hyperglycemia and complications such as ketoacidosis, cardiovascular disease, nephropathy, and retinopathy [7,8]. In 2016, Saudi Arabia was ranked as the second highest in the Middle East and seventh in the world for DM by the World Health Organization (WHO) [9]. The prevalence of DM in Saudi Arabia is alarmingly high, highlighting the importance of screening and following-up patients. Specifically, we must seek to diagnose or screen for depression in people who already have DM [10]. Type-1 diabetes mellitus (T1DM) prevalence in Saudi Arabia varies across regions, ranging from 0.3 to 2.6%. Type-2 diabetes mellitus (T2DM) is more common in Saudi Arabia than T1DM, with a prevalence of 5–15%, and the numbers have been increasing in recent years, due to population urbanization and sedentary lifestyles [11]. Gestational diabetes mellitus (GDM) occurs in approximately 10% of all pregnancies, which is similar to the global averages [12]. Major depression disorder (MDD) is a mood disorder characterized by a depressed mood, loss of interest or pleasure in doing things, changes in sleep pattern (insomnia or sleeping too much), unintended weight gain or loss, feelings of guilt or worthlessness, fatigue or loss of energy, difficulty concentrating, agitation or nervousness, or thoughts of death [13,14]. Both depression and DM are serious threats to global health, affecting ∼9% of the population. The prevalence of DM is rising in all age groups, and it appears that there is a bidirectional etiological relationship between DM and depression. Diabetes and depression are linked and together make healthcare expenses 4.5-times higher for people with diabetes [15,16]. Depression is a common psychological disorder among patients with DM, especially T2DM [17], but there is a lack of knowledge about the association of depression with DM in the Saudi population, specifically in Riyadh. The core symptom used to diagnosis MDD is a diminished or irritable mood for at least 2 weeks [5,13,18]. The symptoms of depression can be different from one patient with DM to another [16]. Undiagnosed depression accompanied by DM can increase the number of sick days and the frequency of hospital admissions, as well as extending hospital stay periods, compared to diabetic patients without depression [19]. Based on gender, diabetic females with depression showed higher mortality rates compared to males, particularly related to cardiovascular diseases, as reported by Pan and co-workers in 2012 [20]. Similarly, a few studies with small sample sizes have been conducted in Saudi Arabia on the prevalence, characterization, and predictors of depression and anxiety among patients with T2DM [21]. A meta-analysis study proposed that diabetic patients with depression showed non-adherence to treatment, poor glycemic control, the development of complications, impaired quality of life, and increased health care costs [22]. In 2016, a study undertaken at the Emergency Eye Hospital Bucharest in Romania reported that “the prevalence rates of depression could be up to three times higher in patients with type one diabetes and twice as high in people with T2DM compared with the general population worldwide” [23]. Patients with T1DM require different treatments and more concerted efforts to manage their disease compared with T2DM. They require frequent monitoring of their glycemia, as well as adjustments to insulin doses, diet, and physical activity. The onset age of patients with T1DM is much earlier than that for T2DM [24]. Depression has been found in diabetic patients receiving oral antidiabetic medications, while no literature is available concerning the association of depression with insulin-dependent T1DM [25]. Both diseases should be recognized and treated on a case-by-case basis. All these steps can help to reduce the incidence of depression and the risk of depression, and help to control DM. Additionally, increasing the awareness of healthcare providers and patients concerning depression in diabetic patients would help to improve overall treatment outcomes. One of the most important methods involves screening for depression in diabetic patients at their regular follow-ups, using screening questionnaires [26].

To ensure a healthy society, it is necessary to prevent, identify, and treat patients’ diseases. However, depression often remains undiagnosed in diabetic patients. Therefore, healthcare providers should be aware of this common comorbidity [16]. The comorbidity of depression in diabetic patients can have critical consequences that may affect the medication regimen, glucose levels (HbA1C), and patient lifestyle [27,28]. Our primary goal in this study was to determine the extent to which T1DM is linked to depressive symptoms. The aims of this study were to determine the prevalence of T1DM in people who also suffer from depression and to establish a link between T1DM, depression, and the risk of developing depression. We also sought to determine the relationship between diagnosis duration in T1DM patients and the presence or absence of depression, as well as the risk of developing depression; the effect of controlled and uncontrolled T1DM on developing depression or the risk of depression; and the effects of other comorbidities on developing depression or the risk of developing depression among Saudi population. The purpose of this study was to estimate the prevalence of T1DM, depression, and depression risk among Saudi patients, as well as to investigate the association between the length of time from diagnosis, glycemic management, and the existence of comorbidities.

## 2. Materials and Methods

This study comprises a retrospective chart review and a quantitative cohort study. We included all patients diagnosed with T1DM between June 2015 and March 2020. The inclusion criteria were Saudi nationality, T1DM, depression, and more than ten years of age. The exclusion criteria were non-Saudi nationality, T2DM, and an age less than ten years. The study variables were categorized based on two main variables. For T1DM, the following were included: 

### 2.1. Duration of Diagnosis

- Newly diagnosed patients (0–1.9 years);
- Two to four years since diagnosis (2–4.9 years);
- Five or more years since diagnosis (≥5 years).

### 2.2. Controlled or Not Controlled

- This was assessed by monitoring patients’ HbA1C and performing fasting blood sugar (FBG) tests.

### 2.3. Comorbidities

- This was assessed by checking if the patient had any other comorbidities, such as cardiovascular disease, liver disease, renal disease, respiratory disease, immunocompromised disease, neurological disease, or cancer in their medical records.

### 2.4. Personal Information

- Marital status—single, married, divorced, or widowed;
- Education level—uneducated, primary and intermediate education, high school education, or bachelor’s degree;
- Body mass index (BMI)—underweight, normal, overweight, or obese;
- Exercise/physical activity—no exercise, once/week, two times/week, three times/week, or more than three times/week.

### 2.5. Depression

- Having depression was determined by looking at the patient’s electronic medical records and dispensed medications;
- The risk of developing depression was determined using PHQ-9 [29].

### 2.6. Ethical Considerations

Ethical approval (E-20-5018) was received from the institutional review board (IRB) at the college of medicine on 9 September 2020. The study was carried out at the diabetes clinics, King Khalid University Hospital (KKUH), Riyadh. The confidentiality and anonymity of the participants’ data were maintained. The obtained data were not used for any other purposes than those of our study. Verbal consent was given by patients after the investigators had introduced themselves, stated the research objectives and the reasons for doing the interview, and asked for consent to participate, without any pressure. A phone interview was undertaken to collect missing information and to assess the patient using the PHQ-9 questionnaire. All the information collected was used for research purposes only.

### 2.7. Data Collection/Data Source

Data were collected by the investigators from the patients’ electronic medical records (eSIHI) held at King Khaled University Hospital, and the investigators used a depression scale (PHQ-9) to assess the risk of developing depression in patients newly and recently diagnosed with T1DM. This depression scale (PHQ-9) consists of two main groups of questions: The first contains nine questions used to assess the presence of symptoms of depression within the last two weeks. The second queries whether these symptoms interfere with the patients’ normal lives. The PHQ-9 is scored between 0 and 27. Scores are classified into five major classes: minimal depression, mild depression, moderate depression, moderate–severe depression, and severe depression. All collected data were protected and analyzed.

### 2.8. Statistical Analysis

Data were entered into, and analyzed using, the Statistical Package for Social Sciences (SPSS) program. Descriptive analysis was performed to determine the frequencies and percentages of the study variables, and these included socio-demographic and T1DM-related variables. The Chi-Square (*X*^2^) test and Fisher’s exact test were used to measure the associations between the study variables. A *p*-value of <0.05 was considered statistically significant.

## 3. Results

**Figure 1:**
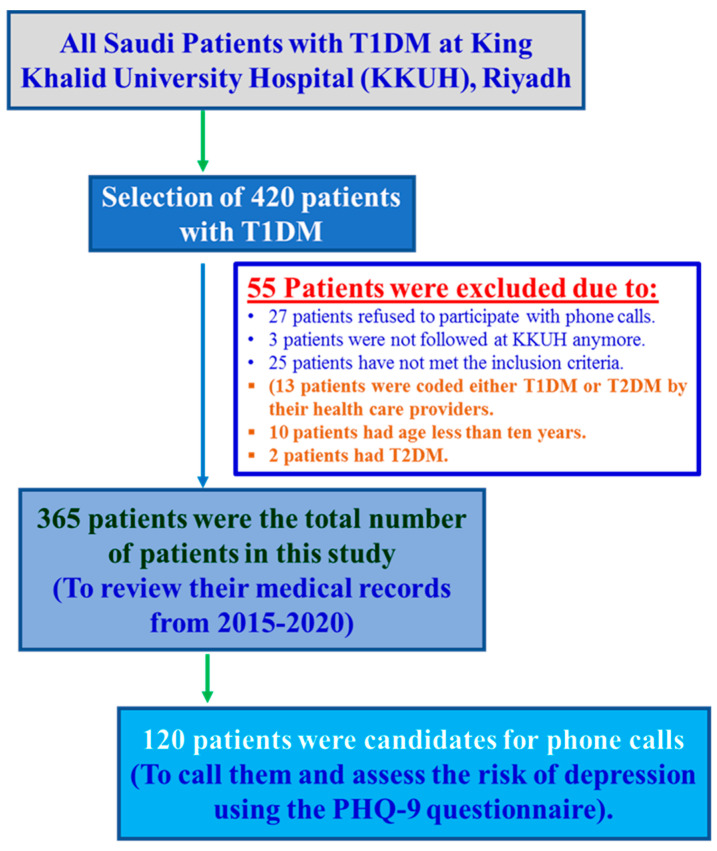
Enrollment of participants/patients in this study.

This study included 365 Saudi patients with T1DM, who were randomly selected from the diabetic clinic at KKUH. The original sample comprised 420 patients; however, around 55 patients were excluded due to their age (less than ten years), nationality (non-Saudi), T2DM diagnosis, having non-classified diabetes (the type of diabetes was not confirmed), no being longer treated at KKUH, or unwillingness to participate (the patients refused to share information over the phone) (Figure 1).

The present study included 167 male (45.75%) and 198 female (54.25%) patients. The patients were grouped into group 1 (10–15 years old), group 2 (16–20 years old), and group 3 (21–24 years old). Group 1 contained 142 patients (38.91%), group 2 contained 147 patients (40.27%), and group 3 contained 76 patients (20.82%) (Table 1). The education level of the patients was classified into four sub-classes: uneducated (0.82%), primary and intermediate (37.53%), high school (27.12%), and bachelor’s degree (34.52%) (Table 1). Patients with a normal body mass index (BMI) constituted 52%, while 21% were underweight, 19% were overweight, and 9% were obese.

Of the 365 patients, 63.30% did not have comorbidities, while 36.71% did (Table 2). The most frequent comorbidities reported were hypertension (HTN), hematological disease, ophthalmological disease, respiratory disease, immunocompromization, neurological disease, psychological disease, dyslipidemia, hypothyroidism, renal disease, gastroesophageal tract disease (GIT), dermatological disease, and others. Furthermore, we classified the patients into four different classes, based on the number of comorbidities (Table 2). The investigators questioned the patients and examined their medical records held within the hospital system (eSIHI), to determine whether they undertook any lifestyle modifications, the type of lifestyle modification, and the frequency of physical activity (if they performed any). Here, 60.82% of the sample undertook no lifestyle modifications, while 39.18% followed some (Table 2). Of this latter 39.18%, the percentage of patients that were on a diet was 32.87%, those undertaking physical activity only was 30.77%, and those both on a diet and undertaking physical activities was 36.26%. We further classified the frequency of physical activity into four sub-groups: once per week (13.54%, 13 patients), two times per week (31.25%, 30 patients), three times per week (16.67%, 16 patients), and more than three times per week (38.54%, 37 patients) (Table 2).

To assess the presence of controlled versus uncontrolled DM in the study sample (N = 365), we attained updated data on HbA1C and fasting blood glucose (FBG). The majority of the sample (72.05%) showed high HbA1C levels (Table 3). Fasting blood level should be measured at less than or equal to 130 mg/dL for diabetic patients. Here, these levels were high (at more than 140 mg/dL) for at least 78.08% of the patients. Furthermore, the investigators classified the study sample into two groups based on the FBG readings: controlled and uncontrolled. The controlled group contained 80 patients (21.9%), while the uncontrolled group contained 285 patients (72.05%) (Table 3). 

The medications used to manage diabetes were insulin (Aspart, Glargine, Humolog Mix, Aspart Protamin with Insulin Lispro, Detemir, NPH, Regular, Tersiba) and some other antidiabetic agents, such as Metformin (in two different dosage forms: extended release and immediate release), Glibenclamide–Metformin, and Liraglutide. Supplementary Table S1 describes the distributions of these medications within the sample. The analytical details of the study sample that used both insulin and hypoglycemic (anti-diabetic) medications are shown in Supplementary Table S1. About 92% (336 patients) of the sample were not on a combination therapy, while 8% (29 patients) were on combination therapy. Three medications were commonly used in this study sample in combination with insulin. The number of patients on metformin with insulin was 25 (86.20%), the number on Glibenclamide–Metformin (5–500 mg) was 2 (6.90%), and the number on Liraglutide was also 2 (6.90%) (Supplementary Table S1). 

The investigators also reviewed the medical records of the sample (365 patients) for diabetic complications and divided the sample into two main groups. The first group without diabetic complications constituted 45%, and the second group with diabetic complications represented 55%. The most common complications were diabetic foot, skin complications, retinopathy, nephropathy, neuropathy, multiple diabetic ketoacidosis (DKA), hypoglycemia, lipo-hypertrophy, recurrent inflammations, and others. Furthermore, we classified the complications according to frequency and type (Supplementary Table S2). Around 7.10% (26 patients) of the sample showed high lipid profiles without a diagnosis of dyslipidemia. About 6.30% (23 patients) had a high A/C ratio and microalbuminuria without treatment; these patients were on neither ACEIs nor ARBs. The number of type-one diabetic patients referred to the psychiatric clinic was 13 (3.6%), and the number of patients who actually attended their psychiatric clinic appointment was four out of these 13.

### 3.1. The Descriptive Statistics for T1DM and Depressed Patients 

The portion of T1DM patients who had been assessed as having depression (according to their medical records) was 6.03%. The number of patients with a positive depression assessment was 17 out of 22 (77.27%), while negative results were derived in five cases (22.73%). The medical records of depressed patients listed Escitalopram, Fluoxetine, and Amitriptyline as medications. Some depressed patients were not followed up at KKUH for the management of their depression; instead, they were followed up at private clinics and hospitals of the Ministry of Health for Psychological Diseases (MOH). For example, many were followed up at Al-Amal Hospital, and most of these were on Escitalopram (47%) (Table 4).

Female patients with T1DM were more frequently found to be depressed (65%; 11 patients out of 17), while the males numbered only six (35%) (Supplementary Table S3). The duration of diagnosis with depression was classified into three categories. The first category was less than two years (0–1.9 years). The second category was two to four years (2–4.9 years). The third category was greater than or equal to five years (>=5 years). The majority of depressed patients with T1DM had been diagnosed with depression within the last two years (approximately 71%). The age groups of diabetic patients diagnosed with depression, as well as their material status, educational level, and descriptive analytic details are shown in Supplementary Table S3. 

### 3.2. The Testing Hypothesis for T1DM and Depressed Patients (n = 17) and Relationship between T1DM and Depression (n = 22)

The analysis included 22 respondents who had undergone documented depression assessment, and these were subjected to medical record reviews, while the rest were excluded, as shown in Supplementary Table S3. The independent variables were gender, age, educational level, marital status, BMI, comorbidities, lifestyle modification, type of lifestyle modification, and frequency of physical activity. In contrast, the outcome variables comprised the results of the depression assessment. The results revealed that educational level was most significantly associated with depression ((*p* < 0.05) *X*^2^ (3, N = 22) = 15.94, *p* = 0.001), followed by lifestyle modifications (Supplementary Table S4), complications and diagnosis (Supplementary Table S5), controlled DM (Supplementary Table S6), and comorbidities (Supplementary Table S7) (*p* < 0.05). The rest of the variables did not show any statistically significant relationship/association with depression (*p* > 0.05). In total, 5% (17 out of 365 patients) of the overall sample were diagnosed with depression, and the percentage of the sample diagnosed by a healthcare provider at a hospital was 77.27% (17 out of 22 patients). The prevalence of T1DM with a risk of developing depression among the Saudi population was found to be 62.50% (75 out of 120 patients).

### 3.3. The Effect of Controlled DM and Uncontrolled DM on Developing Depression among the Saudi Population

The effects of glycemic control and non-control on the likelihood of developing T1DM with depression were studied using target readings for both HbA1C and FBG. All patients with T1DM and depression (n = 17) had uncontrolled DM (glycemic no-control) according to the HbA1C readings. In total, three patients showed HbA1C in the range of 7.10–8.90%, and 14 patients showed readings greater than or equal to 9% (Supplementary Table S3). According to the FBG reading, 15 out 17 patients had uncontrolled DM, and two out of 17 patients had controlled DM. The results indicated a relationship between the control of DM and having depression among the Saudi population. This result is supported by the significant relationship found between these two variables using the HbA1C (*X*^2^ (1, N = 22) = 0.0007, *p* = 0.05) and FBG readings (*X*^2^ (1, N = 22) = 0.0008, *p* = 0.05) (Supplementary Table S6).

### 3.4. Effects of Other Comorbidities on Having Depression among the Saudi Population with T1DM and Depression (n = 22)

The comorbidity variable was divided into two. Accordingly, the test was able to successfully show a statistically significant relationship between the two variables (*X*^2^ (1, N = 22) = 4.0903, *p* = 0.04), as shown in Supplementary Table S7.

### 3.5. Descriptive Statistics for T1DM Patients and Their Risk of Developing Depression (n = 120) 

The investigators randomly selected 120 patients out of the original 365 and called them to assess their risk of developing depression (assessments were performed using the PHQ-9 questionnaire in October–November 2020). All but 27 of the participants agreed to participate in this study (some of these were juveniles whose parents refused to let them participate in this study). A total of 120 patients were interviewed in the sample. The frequency of patients found to be at risk of developing depression was 75 out of 120 (62.50%), while 45 (37.50%) were not at risk. The depression scores allocated to the 75 patients at risk of depression were divided into three groups. Those at risk of developing mild depression comprised 62.67%, those at risk of moderate depression—36%, and those at risk of moderate to severe depression—1.33% (Table 5).

A descriptive statistical analysis was performed on the 120 patients who were not assessed (Supplementary Table S8). These included 59 male and 61 female patients. Their distribution was based on the outcomes of assessments, which classified them into two groups; the negative group contained 45 patients, with 19 males (15.83%) and 26 females (21.67%); and the positive group contained 75 patients, consisting of 40 males (33.33%) and 35 females (29.20%) (Supplementary Table S8). The most common age range in the negative group was 10–15 years (22 patients; 18.33%), and in the positive group it was 16–20 years (32 patients; 26.67%). Most patients showed a normal body mass index (BMI) in both groups. The frequency of comorbidities was classified into two groups based on the results of the PHQ-9 questionnaire: negative and positive. The number of patients with comorbidities in the negative group was 20 (27.03%)—18 with one comorbidity and two with two comorbidities. The positive group contained 54 patients with comorbidities (72.97%)—36 with one comorbidity, 14 with two comorbidities, two with three comorbidities, and two with more than three comorbidities. In total, 33 patients who had had T1DM for more than five years showed a negative result according to the PHQ-9 questionnaire, while 65 patients who had been diagnosed with T1DM for more than five years showed a positive result (Supplementary Table S8). The numbers of those in the negative and positive groups who performed physical activities each week are shown in Supplementary Table S8. The investigators found that most of the negative group performed physical activities more than three times per week, while most of the positive group undertook physical activities two times per week. 

Based on the HbA1c and FBG readings, we classified the samples into controlled and uncontrolled DM. In the negative group, two patients out of 45 (2%) were under control, while 43 (36%) were not controlled, based on their HbA1c readings. In the positive group, that nine out of 75 patients were controlled (7.50%), and 66 (55%) were not controlled. The frequencies of complications are shown in Supplementary Table S8. More than half of the negative and positive groups took insulin in the form of Aspart with Glargine (44 out of 45 (36.67%) and 71 out of 75 (59.20%), respectively). In the negative group, those using a combination therapy of insulin and other anti-diabetic medications numbered two out of 45 (1.67%). Two patients on a Metformin and insulin combination therapy showed no risk of developing depression (Supplementary Table S8).

### 3.6. The Hypothesis Testing for T1DM and Patients at Risk for Depression (n = 120) and the Relationship between T1DM and Risk for Depression (N = 120)

The outcome variable was the risk of developing depression. The results reveal that the variables of controlled/uncontrolled DM, the presence of comorbidities, and using Metformin were significantly associated with the risk of developing depression (*p*-value < 0.05), as shown within the tables (Supplementary Table S9).

### 3.7. The Effect of Controlled DM and Uncontrolled DM on Developing Depression among Saudis (n = 120): The Effects among T1DM and Patients at Risk for Depression

The FBG readings show that 14 patients were controlled and 61 patients were not controlled (Supplementary Table S8). No relationship could be observed between T1DM and the risk of developing depression based on the Hb1AC readings (*X*^2^ (1, N = 120) = 1, *p* = 0.05) as compared to the FBG readings (*X*^2^ (1, N = 120) = 8, *p* = 0.0047) (Supplementary Table S9).

## 4. Discussion

The risk of developing a mental illness such as depression at some point throughout life is about 50%; such conditions can lead to a reduction in employment, productivity, and wages [16]. Depression also leads to poor self-care behavior, which affects the treatment outcomes of DM. In addition, the occurrence of depression in diabetic patients may lead to more complications related to diabetes [30]. The complications of DM can be classified into macrovascular and microvascular, such as ophthalmological, renal, cardiovascular, neurological, and musculoskeletal issues [21]. It is well established that depression in patients with other comorbidities is associated with the dysregulation of the hypothalamic–pituitary–adrenal axis, the activation of the sympathetic nervous system, and the production of proinflammatory and procoagulation markers [31,32]. These inflammatory responses associated with depression may also lead to macrovascular and microvascular consequences in T2DM patients [33]. The current study comprised three sections. The first section addressed a whole sample of diabetic patients (N = 365), to determine the number with depression. The second section addressed all diabetic patients who had previously received a depression assessment (n = 22). The third section concerned a random sample (n = 120). Patients with diabetes who also have a lack of glycemic, diabetic complications, DM with multiple comorbidities, and lack lifestyle adjustments, and/or who are on a combination of anti-diabetic drugs were shown to be at a higher risk of developing depression in the current study. Based on these findings, we implemented depression assessment and screening procedures for all diabetes patients with at least one of these risk factors.

Of the diabetic patients with confirmed depression (n = 17), 65% were females and 35% were males. Depression was found to be more common among females than males when diagnosed by healthcare providers at the hospital. In contrast, males showed a higher risk of depression based on the results of the PHQ-9 questionnaire applied to a random sample (n = 120). The present results are consistent with those of Alkot and co-workers [34]. Educational level was determined to be a statistically significant risk factor for diabetic patients being confirmed with depression (n = 17). Patients with bachelor’s degrees (53%) showed the greatest labels of confirmed depression compared to patients with other educational levels. Al-Hunayani’s study states that a low educational level is a statistically significant risk factor for developing depression among T2DM patients [35]. Our findings regarding the effects of educational level differ from those of the previous study (Al-Hunayani study). Our study also showed that those with a bachelor’s degree and T1DM were at a higher risk of developing depression. This divergence may relate to the different sample groups used (T2DM vs. T1DM); geographical variations (Arar vs. Riyadh); and differences in culture, personal behaviors, and beliefs. Moreover, Alkot et al. reported that BMI—especially obesity—represents a risk factor for developing depression [34]. Regarding the effects of BMI in relation to developing depression among diabetic patients, our results are not in agreement with those of Alkot M et al. Here, most depressed patients with T1DM and diabetic patients with a risk of depression had normal BMI values, of 59% and 30.83%, respectively. This may be due to the differences between study populations. The present study only included T1DM patients, while Alkot M et al. reported on all types of DM. Additionally, it may be related to the different geographical areas assessed. 

Glycemic non-control is an essential risk factor for having or developing depression among diabetic patients [36]. The literature published on DM in general, and T2DM in particular, states that poor glycemic control may lead to physical inactivity, non-adherence to either lifestyle modifications or anti-diabetic medications, and poor quality of life, resulting from the development of multiple complications [37,38]. Several studies have been conducted at hospitals in different regions of Saudi Arabia, with different objectives and varied sample sizes. Most of these focused on T2DM and DM more generally. The study designs were mostly cross-sectional. A study was carried out by Al-Hunayani et al. on the prevalence of and risk factors contributing to depression among T2DM patients in Arar city [35]. Another study conducted by Alkot M et al. was performed in the Makkah region, to determine the prevalence of depression and associated factors among DM patients [34]. A further study conducted by Al-Ghamdi et al. in Jeddah between 2002 and 2003 determined the prevalence of depression among diabetic patients [39]. Assessments of the relation between depression and different types of DM were carried out at Al-Solimania Primary Health Care Center in Riyadh over four months, with a sample size of 100 patients, by the authors of [40]. Our results agree with those of the reports mentioned above. 

The findings of the current study showed a statistically significant relationship between diabetic complications and depression. These findings are in line with the results of previous studies [35,39]. Al-Ghamdi et al. showed that 34% of diabetic patients with depression were at a higher risk of developing complications, including micro- and macrovascular complications [39]. The most common diabetic complications reported by Al-Ghamdi et al. were related to microvasculature and retinopathy, which further supports our results. Furthermore, the depressed patients with T1DM assessed in the current study showed complications related to nephropathy, multiple attacks of DKA, hypoglycemia, and lipohypertrophy. The present results also clarified the association of depression with an increased number of complications, which is consistent with the results of de Groot and co-workers [30]. Diabetic patients with depression generally showed one to two diabetes-related complications (Supplementary Table S3). About 70.59% of the T1DM patients with confirmed depression (n = 17) had comorbidities. Here, 45% of a random sample (n = 120) had comorbidities associated with T1DM, and were at risk of developing depression (Supplementary Table S8). A statistically significant relationship was found between the presence of comorbidities and having depression among T1DM patients (Supplementary Table S7). 

Physical activity is necessary for improving the health of all patients, regardless of their age [9]. Abdulaziz Al Dawish and his colleges reported on the prevalence of physical activity among Saudi adults, finding that most of the Saudi population are physically inactive and have higher incidences of T2DM than other types of DM, due to this limited physical activity [9]. The current study reports that treating T1DM necessitates physical activity, as much as T2DM. The variables of glycemic control, psychological emotional state, BMI, and others were better in patients undertaking physical activity at least three times per week. Patients undertaking physical activity more than three times per week had a low risk of developing depression (Supplementary Table S8). However, diabetic patients are at risk of developing depression and should be encouraged to participate in physical activities at least three times a week. Our results also show that a diabetic patient with limited physical activity is more likely to be at risk of depression. Al-Hunayni et al. reported that poor compliance with physical activities and diet regimen are significantly associated with depression, which agrees with the present results [35].

Some diabetic patients with confirmed depression were on a combination therapy (insulin and Metformin) (Supplementary Table S3). The number of diabetic patients on a combination therapy with a risk of depression was nine, and seven out of these nine were on metformin and needed to be assessed for depression (Supplementary Table S8). Al-Hunayni showed that low educational level, insufficient income, and long duration of DM are risk factors for developing depression among T2DM patients [35]. This diverges from the results of our study, which were related to a different sample, with differences in geographical region, culture, and other factors. The current study agrees with the findings regarding duration of diagnosis, but here this finding was not statistically significant. Most diabetic patients with confirmed depression (n = 17) had been diagnosed with DM over five years ago. This study’s findings did not agree with this regarding low educational level. Due to the COVID-19 pandemic, no recent laboratory test data were available for most patients; there was also a lack of good follow-up procedure amongst the patients compared to the pre-pandemic period, and patients generally avoided hospital visits. These were the limitations of the present study, which as primarily related to fear of catching COVID-19, as well as other unknown reasons. The sample size was small because very few patients were assessed for depression by the healthcare providers at the hospital. Some patients were also not willing to participate.

## 5. Conclusions and Limitations

To overcome the negatives consequences of undiagnosed depression, screening is recommended for patients with DM who have multiple comorbidities, glycemic non-control, diabetic complications, and no lifestyle modifications, as well as those on a combination therapy with Metformin. Therefore, it is important for the healthcare providers of patients with DM to monitor for signs and symptoms of psychological distress, in order to provide timely interventions aimed at reducing the risks of the development or worsening of psychological disorders such as depression.

## Figures and Tables

**Table 1:** Distribution of socio-demographic variables (N = 365).

Variables	Categories	Frequency	%
Gender	Male	167	45.75
	Female	198	54.25
	Total	365	100
Age in years	10–15 years	142	38.91
	16–20 years	147	40.27
	21–24 years	76	20.82
	Total	365	100
Marital status	Single	363	99.45
	Married	2	0.55
	Total	365	100
Educational level	Uneducated	3	0.82
	Primary and intermediate education	137	37.53
	High school education	99	27.12
	Bachelor’s education	126	34.52
	Total	365	100.0
Body Mass Index (BMI)	Underweight (X < 18.5)	76	21.00
	Normal (X = 18.5–24.9)	188	52.00
	Overweight (X = 25–29.9)	69	19.00
	Obese (X ≥ 30)	32	9.00
	Total	365	100

**Table 2:** Distribution of T1DM-related variables (N = 365).

Variables	Categories	Frequency	%
Comorbidities	No comorbidity	231	63.30
	Yes comorbidity	134	36.71
	Total	365	100.0
Yes comorbidity	One comorbidity	66	49.30
	Two comorbidities	40	29.90
	Three comorbidities	19	14.20
	More than three comorbidities	9	6.71
	Total	134	100.00
The duration of diagnosis	Less than 2 years (0–1.9 year)	5	1.37
	2–4.9 years	66	18.08
	Greater than or equal to 5 years (≥5 years)	294	80.55
	Total	365	100.00
Lifestyle modification	No	222	60.82
	Yes	143	39.18
	Total	365	100.00
The type of lifestyle modification that they undertook	Diet	47	32.87
	Exercise	44	30.77
	Both of diet and exercise	52	36.36
	Total	143	100.00
Physical activity	No exercise or any physical activity	269	73.70
	Yes, exercise or other physical activity	96	26.30
	Total	365	100.0
The frequency of exercise	Once a week	13	13.54
	Two times a week	30	31.25
	Three times a week	16	16.67
	More than three times a week	37	38.54
	Total	96	100.00

**Table 3:** Description of the study sample according to controlled or uncontrolled HbA1C and FBG.

HbA1C Reading	Count	Percentage (%)
Controlled DM (HbA1C ≤ 7%)	18	4.93
Uncontrolled DM		
HbA1C 7.1–8.9%	84	23.01
HbA1C ≥ 9%	263	72.05
Total	365	100
FBG reading	Count	Percentage (%)
Controlled DM (X ≤ 139 mg/dL)	80	21.90
Uncontrolled DM (X ≥ 140 mg/dL)	285	78.10
Total	365	100

**Table 4:** Distribution of the study sample (N = 365) according to their having been subjected to depression assessment, and descriptions of anti-depressant medications.

Variables	Categories	Frequency	%
Was the T1DM patient assessed for depression?	No	343	93.97
	Yes	22	6.03
	Total	365	100
The results of depression assessment (n = 22)	Negative (−ve)	5	22.73
	Positive (+ve)	17	77.27
	Total	22	100
Anti-depressant medications used by this sample (n = 17)	Escitalopram	8	47
	Fluoxetine	5	29
	Amitriptyline	4	24
	Total	17	100

**Table 5:** Risk of developing depression among T1DM patients (n = 120).

Variables	Categories	Frequency	%
Using PHQ-9 questionnaire			
The risk of developing depression	No	45	37.50
	Yes	75	62.50
	Total	120	100
The score of depression risk	Risk of mild depression	47	62.67
	Risk of moderate depression risk	27	36.00
	Risk of moderate to severe depression	1	1.33
	Total	120	100

## Data Availability

The data generated in the study are clearly presented in the manuscript and in the Supplementary file.

## Supplementary Materials

The following supporting information can be downloaded at: https://www.mdpi.com/article/10.3390/jpm13040654/s1, Table S1: Description of the study sample according to DM medications. Table S2: Description of the study sample (N = 365) according to DM complications. Table S3: Descriptive analysis for diabetic patients diagnosed with depression and duration of diagnosis. Table S4: The results of Chi-Square tests and Fisher’s exact tests to identify the association of T1DM variables and depression (n = 22). Table S5: The results of Chi-Square tests (*X*^2^) for duration of diagnosis and depression (N = 22). Table S6: The results of Fisher’s exact tests for controlled/uncontrolled DM and depression (N = 22). Table S7: The results of Chi-Square tests (*X*^2^) for comorbidities and depression (N = 22). Table S8: The descriptive statistics for T1DM patients who were assessed for depression by phone call/interview (n = 120). Table S9: The results of the Chi-Square test (*X*^2^) for duration of diagnosis and depression risk (N = 22). The results of Fisher’s exact test for controlled/uncontrolled DM and depression risk (N = 120). The Chi-Square test for controlled/uncontrolled DM and depression risk (N = 120). The Chi-Square test for comorbidities and DM with depression risk (N = 120). The Chi-Square test for using Metformin as adjuvant therapy in T1DM patients with depression risk (n = 10).
