# Supplementary material for: The Association between Type-1 Diabetes Mellitus and Risk of Depression among Saudi Patients: A Cross-Sectional Study
Source: J Pers Med. 2023 Apr 11;13(4):654. doi: 10.3390/jpm13040654 (PMC10146660; doi:10.3390/jpm13040654)
Supplement: Supplementary file 1 [file jpm-13-00654-s001.zip › jpm-2300705-supplementary.pdf]

List of supplementary Tables:

Supplementary Table S1: The description of the study sample according to the DM medications.

Variables	Categories	Frequency	%
**Type of Insulin**	Insulin Aspart, Glargine	343	94
	Insulin Aspart, Tresiba	7	1.92
	Insulin Aspart, Detemir	4	1.10
	Insulin Aspart	6	1.64
	Others	5	1.36
	**Total**	**365**	**100**
**Type of Insulin**	Basle-Bolus regimen	354	97.02
	Continuous pumping of insulin	9	2.5
	others	2	0.55
	**Total**	**365**	**100**
**Using of insulin pump/infusion**	No	356	97.5
	Yes	9	2.5
	**Total**	**365**	**100.0**
**Using the combination of insulin and other anti-diabetic medications** (oral or injection form)	No	336	92
	Yes	29	8
	**Total**	**365**	**100**
**The anti-diabetic medications**	Metformin	25	86.2
	Glibenclamide-Metformin	2	6.9
	Liraglutide	2	6.9
	**Total**	**29**	**100**

(**Supplementary Table S2**)**:** The description of the study sample (N=365) according to the DM complications**.**

Variables	Categories	Frequency	%
**Complications**	No	167	45.80
	Yes	198	54.24
	**Total**	**365**	**100.0**
**Type of complications**	Skin complication	9	3
	Diabetic foot	4	1
	Retinopathy	27	10
	Neuropathy	6	2
	Nephropathy	43	15
	DKA	80	28
	Hypoglacemia	64	23
	Lipohypertrophy	47	17
	Recurrent inflammation	4	1
**The Diabetic complications based on the frequency of having complications (n=198)**	One complication	133	67.20
	Two complications	48	24.24
	Three complications	13	6.56
	More than three complications	4	2.02
	**Total**	**198**	**100.0**

(**Supplementary Table S3**): The descriptive analysis for diabetic patients whose diagnosed with depression and duration of diagnosis.

Variables	Categories	Frequency	%
**Type one diabetic patients with depression (n=17)**			
**Gender**	Male	6	35
	Female	11	65
	**Total**	**17**	**100.0**
**Age**	10-15 years	6	35.3
	16-20 years	6	35.3
	21-24 years	5	29.4
	**Total**	**17**	**100.0**
**Material status**	Single	17	100.0
	Married	0	0
	**Total**	**17**	**100.0**
**Educational level**	Primary and intermediate education.	6	35
	High school education	2	12
	Bachelor education.	9	53
	**Total**	**17**	**100.0**
**BMI**	Underweight (X<18.5)	4	24
	Normal (X=18.5-24.9)	10	59
	Overweight (25-29.9)	3	18
	Obese. (X>= 30)	0	0
	**Total**	**17**	**100.0**
**Comorbidity**	No	5	29.41
	Yes	12	70.59
	**Total**	**17**	**100.0**
**Duration of diagnosis**	2-4 years of diagnosis.	2	11.76
	Greater than or equal to 5 years of diagnosis.	15	88.24
	**Total**	**17**	**100.0**

**Followed the lifestyle modifications**	No	13	76.5
	Yes	4	23.5
	**Total**	**17**	**100.0**
**Type of lifestyle modifications (n=4)**	Diet ONLY.	1	25.0
	Exercise ONLY.	2	50.0
	Both of diet and exercise.	1	25.0
	**Total**	**4**	**100.0**
**The frequency of exercise (n=3)**	Once a week	1	33.0
	Two times a week	1	33.0
	Three times a week	1	33.0
	**Total**	**3**	**100.0**
**HbA1C reading**	A. **Controlled DM (HbA1C ≤ 7%)**	0	0
	B. **Uncontrolled DM:**	**17**	**100.0**
	▪ HbA1C 7.1% - 8.9%	3	18.0
	▪ HbA1C ≥ 9 %	14	82.0
	**Total**	**17**	**100.0**
**Fasting reading (FBG)**	A. Controlled DM (X ≤ 139 mg/dL)	2	12
	B. Uncontrolled DM (X≥ 140 mg/dL)	15	82
	**Total**	**17**	**100.0**
**Complications**	No	3	17.65
	Yes	14	82.35
	**Total**	**17**	**100.0**
**The frequency of complications**	One complication	7	50.0
	Two complications	7	50.0
	**Total**	**14**	**100.0**
**The most common complications among T1DM with depression**	Skin complication	1	5.0
	Retinopathy	2	10.0
	Nephropathy	6	29.0
	DKA	6	29.0

	Hypoglacemia	3	14.0
	Lipohypertrophy	3	14.0
	**Total**	**21**	**100.0**
**Using the combination of therapy (insulin and other anti-diabetic medications)**	No	16	94.0
	Yes	1	6.0
	**Total**	**17**	**100.0**
**Using insulin pump (infusion)**	No	17	100.0
	Yes	0	0
	**Total**	**17**	**100.0**
**The duration of diagnosis by depression (n=17)**	Less than two years (0-1.9 years)	12	71
	From two to four years (2-4.9 years)	3	18
	Greater than or equal to five years (≥5 years).	2	12
	**Total**	**17**	**100.0**

Supplementary Table S4: The results of chi-square test and fisher exact test to identify the association of T1DM variable and depression (n=22)**.**

Variable	Categories	Depression			X^2 value	df	P value
		-ve	+ve	Total			
**Educational level (Chi square test)**	Uneducated	1	0	1	**15.944**	**3**	**.001**
	Primary and intermediate education	0	6	6			
	High school education	4	2	6			
	Bachelor education	0	9	9			
	**Total**	**5**	**17**	**22**			
**Lifestyle modifications** (Fisher exact test)	Not followed	5	13	18	**0.0048**	**1**	**0.05**
	Yes followed	0	4	4			
	**Total**	**5**	**17**	**22**			
**Complications (Chi square test)**	With complications	1	14	15	**6.9241**	**1**	**0.0085**
	Without complications	4	3	7			
	**Total**	**5**	**17**	**22**			

Supplementary Table S5: The results of Chi square test (X^2) for duration of diagnosis and depression (N = 22).

Variable	Categories	Depression			X^2 value	df	P value
		-Ve	+Ve	Total			
Duration of diagnosis	2-4 years	1	2	3	0.206	1	0.650
	Greater than or equal 5 years	4	15	19			
	Total	5	17	22			

Supplementary Table S6: The results of fisher exact test for controlled/uncontrolled DM and depression (N = 22).

Variable	Categories	Depression			X^2 value	df	P value
		-Ve	+Ve	Total			
**HbA1C**	Controlled	4	0	4	0.0007	1	0.05
	Uncontrolled	1	17	18			
	**Total**	**5**	**17**	**22**			
**FBG**	Controlled	5	2	4	0.0008	1	0.05
	Uncontrolled	0	15	18			
	**Total**	**5**	**17**	**22**			

Supplementary Table S7: The results of Chi square test (X^2) for comorbidities and depression (N = 22).

Variable	Categories	Depression			X^2 value	df	P value
		-Ve	+Ve	Total			
**Comorbidities**	With comorbidities	1	12	13	4.0903	1	0.04313
	Without comorbidities	4	5	9			
	**Total**	**5**	**17**	**22**			

Supplementary Table S8: The descriptive statistical for T1DM whose had the assessment of depression by phone calling/interview (n=120).

Variables	Result of assessment	Categories	Frequency	%
- **Gender**	-ve (n=45)	Male	19	15.83
		Female	26	21.67
	+ve (n=75)	Male	40	33.33
		Female	35	29.2
	**Total**		**120**	**100.0**
- **Age**	-ve (n=45)	10-15 years	22	18.33
		16-20 years	14	11.67
		21-24 years	9	7.5
	+ve (n=75)	10-15 years	23	19.67
		16-20 years	32	26.67
		21-24 years	20	16.67
	**Total**		**120**	**100.0**
- **BMI**	-ve (n=45)	Underweight	14	11.67
		Normal	22	18.33
		Overweight	7	5.8
		Obese	2	1.67
	+ve (n=75)	Underweight	11	9.2
		Normal	37	30.83
		Overweight	15	12.5
		Obese	12	10
	**Total**		**120**	**100.0**
- **Comorbidity:**	-ve (n=45)	No	25	20.83
		Yes	20	16.67
	+ve (n=75)	No	21	17.50
		Yes	54	45.0
	**Total**		**120**	**100.0**

- **The frequency of comorbidity:**	-ve (n=20)	One comorbidity	18	**24.32**
		Two comorbidities	2	**2.70**
		Three comorbidities	0	**0.0**
		More than three comorbidities	0	**0.0**
	+ve (n=54)	One comorbidity	36	**48.64**
		Two comorbidities	14	**18.91**
		Three comorbidities	2	**2.70**
		More than three comorbidities	2	**2.70**
	**Total**		**74**	**100.0**
- **The duration of diagnosed by T1DM**	-ve (n=45)	0-1.9 years	0	0
		2-4 years	12	10
		>=5 years	33	27.5
	+ve (n=75)	0-1.9 years	1	1
		2-4 years	9	7.5
		>=5 years	65	54.2
	**Total**		**120**	**100.0**
- **The lifestyle modification followed**	-ve (n=45)	No	21	17.5
		Yes	24	20
	+ve (n=75)	No	47	39.17
		Yes	28	23.33
	**Total**		**120**	**100.0**
- **Type of lifestyle which they are followed (n=52):**	-ve (n=24)	Diet ONLY.	4	7.69
		Exercise ONLY.	8	15.38
		Both of diet and exercise.	12	23.1
	+ve (n=28)	Diet ONLY.	14	27.0
		Exercise ONLY.	10	19.23
		Both of diet and exercise.	4	8.0
	**Total**		**52**	**100.0**

- **The frequency of physical activity (n=34):**	-ve (n=20)	once/week.	1	3.0
		Two times/week.	8	23.53
		Three times/week.	1	3.0
		More than three times/week.	10	29.41
	+ve (n=14)	once/week.	1	3.0
		Two times/week.	8	23.52
		Three times/week.	0	0.0
		More than three times/week.	5	14.71
	**Total**		**34**	**100.0**
- **HbA1C reading:**	-ve (n=45)	Controlled DM (HbA1C ≤ 7%)	2	**2.0**
		Uncontrolled DM (36%):		
		HbA1C 7.1% - 8.9%	14	12.0
		HbA1C ≥ 9 %	29	24.0
	+ve (n=75)	Controlled DM (HbA1C ≤ 7%)	4	3.0
		Uncontrolled DM (59%):		
		HbA1C 7.1% - 8.9%	19	16.0
		HbA1C ≥ 9 %	52	43.0
	**Total**		**120**	**100.0**
- **Fasting reading (FBG):**	-ve (n=45)	Controlled DM (X ≤ 139 mg/dL)	15	12.5
		Uncontrolled DM (X≥ 140 mg/dL)	30	25.0
	+ve (n=75)	Controlled DM (X ≤ 139 mg/dL)	9	7.5
		Uncontrolled DM (X≥ 140 mg/dL)	66	55.0
	**Total**		**120**	**100.0**
- **Complications:**	-ve	No	27	22.5

	(n=45)	Yes	18	15.0
	+ve (n=75)	No	37	30.83
		Yes	38	31.67
	**Total**		**120**	**100.0**
**The frequency of complications:**	-ve (n=18)	One complication	11	61.0
		Two complications	5	28.0
		Three complications	2	11.0
		More than three complications	0	0.0
	**Total**		**18**	**100.0**
	+ve (n=38)	One complication	24	63.0
		Two complications	9	24.0
		Three complications	4	11.0
		More than three complications	1	3.0
	**Total**		**38**	**100.0**
- **Type of insulin:**	-ve (n=45)	Insulin Aspart, Glargine.	44	36.67
		Insulin Aspart,Tresiba.	1	0.83
		Insulin Aspart, Detemir.	0	0.0
		Insulin Aspart.	0	0.0
		Others.	0	0.0
	+ve (n=75)	Insulin Aspart, Glargine.	71	59.20
		Insulin Aspart,Tresiba.	1	0.83
		Insulin Aspart, Detemir.	0	0.0
		Insulin Aspart.	2	1.67
		Others.	1	0.83

	**Total**		**120**	**100.0**
- **Use the combination of insulin and anti-diabetic medications**	-ve (n=45)	No	43	35.83
		Yes	2	1.67
	+ve (n=75)	No	66	55.0
		Yes	9	8.0
	**Total**		**120**	**100.0**
- **The frequency of anti-diabetic medications (n=11)**	-ve (n=2)	Metformin	2	18.2
		Glibenclamide-Metformin	0	0.0
		Liraglutide	0	0.0
	+ve (n=8)	Metformin	7	64.0
		Glibenclamide-Metformin	1	9.1
		Liraglutide	1	9.1
	**Total**		**11**	**100.0**

Supplementary Table S9: The results of Chi square test (X^2) for duration of diagnosis and depression risk (N = 22). The fisher exact test for controlled/uncontrolled DM and depression risk (N = 120). The Chi square test for controlled/uncontrolled DM and depression risk (N = 120). The Chi square test for comorbidities and DM with depression risk (N = 120). The Chi-square test for using metformin as adjuvant therapy at T1DM patients with depression risk (n = 10).

**Variable**	**Categories**	**The risk of developing depression**			**X^2 value**	**df**	**P value**
		No	Yes	Total			
Duration of diagnosis	Less than four years (0-4.9 years)	12	10	63	3.3395	1	0.0676
	More than five years (≥5years)	33	65	57			
	Total	45	75	120			
**Variable**	**Categories**	**The risk of developing depression**			**X^2 value**	**df**	**P value**
		No	Yes	Total			
HbA1C	Controlled	2	4	6	1	1	0.05
	Uncontrolled	43	71	114			
	**Total**	45	75	120			
**Variable**	**Categories**	**The risk of developing depression**			**X^2 value**	**df**	**P value**
		No	Yes	Total			
FBG	Controlled	15	9	24	8	1	0.00467
	Uncontrolled	30	66	96			
	**Total**	45	75	120			
Variable	**Categories**	**The risk of developing depression**			**X^2 value**	**df**	**P value**
		-Ve	+Ve	Total			
Comorbidities	With comorbidities	20	54	74	9.0341	1	0.00265
	Without comorbidities	25	21	46			
	**Total**	45	75	120			
Variable	**Categories**	**Have the risk of depression**			**X^2 value**	**df**	**P value**
		No	Yes	Total			
Metformin as adjuvant therapy	Controlled DM	1	0	1	6.5689	1	0.05
	Uncontrolled DM	1	8	9			
	**Total**	2	8	10			
